# A Preliminary Evaluation of the Prognostic Role of HER-2 and HER-3 Immunohistochemical Expression in Canine Melanomas

**DOI:** 10.3390/ani14101400

**Published:** 2024-05-07

**Authors:** Francesca Parisi, Luigi Aurisicchio, Arianna Pecorari, Alessandro Poli, Francesca Millanta

**Affiliations:** 1 Dipartimento di Scienze Veterinarie, Università di Pisa, Viale delle Piagge 2, 56124 Pisa, Italy; aripecorari@gmail.com (A.P.); alessandro.poli@unipi.it (A.P.); francesca.millanta@unipi.it (F.M.); 2 Evvivax Srl, Via Castel Romano 100, 00128 Rome, Italy; aurisicchio@evvivax.com

**Keywords:** cancer, canine melanoma, HER-2, HER-3, immunohistochemistry, immunotherapy

## Abstract

**Simple Summary:**

Epidermal growth factor receptors (EGFRs) have been suggested as prognostic markers and potential therapeutic targets in several neoplasms in both human and veterinary medicine, among these is human cutaneous melanoma. The aim of this study was to evaluate the immunohistochemical expression of human epidermal growth factor receptors HER-2 and HER-3 in canine melanomas and correlate their expression to clinicopathological parameters. HER-2 expression was observed in 48.6% of the samples and HER-3 expression in 18.9%, while the coexpression of the two receptors was detected in 13.5% of the samples. The expression of both markers was significantly correlated to the presence of ulceration in oral melanomas. A statistically significant association (*p* < 0.05) was observed between the expression of HER-2 and HER-3 and the presence of ulceration in oromucosal tumors. This work confirmed the expression of HER-2 and HER-3 in canine melanomas. Further studies are necessary to investigate the prognostic and therapeutic role of EGFR family receptors in canine melanomas.

**Abstract:**

Canine melanoma is a malignant and aggressive neoplasm showing clinical, histological, and molecular features similar to the human counterpart. In human medicine, epidermal growth factor receptors (EGFRs) have already been suggested as prognostic markers and potential therapeutic targets in cutaneous melanoma. The aim of this study was to evaluate the expression of HER-2 and HER-3 in canine melanomas by immunohistochemistry and correlate their expression to the clinicopathological parameters of the examined tumors. Thirty-seven canine melanoma samples were recruited. Data regarding signalment and clinical parameters were also collected. The population was composed of 18 cutaneous, 16 oral/mucosal, and three digital/foot pad melanomas. Histopathological investigations were carried out to analyze histological type, ulceration, and mitotic count. On each sample, immunohistochemistry was performed using an anti-Melan-A or anti-Melanoma antigen, i.e., anti-HER-2 and anti-HER-3 antibodies. HER-2 and HER-3 positivity were classified using already established scoring criteria and a statistical analysis was carried out. The results highlighted that HER-2 expression was observed in 48.6% of the samples and HER-3 expression in 18.9%. The highest HER 2 score (3+) was recorded in 16.2% of the samples, while the coexpression of the two receptors was detected in 13.5% of the samples. A statistically significant association (*p* < 0.05) was observed between the expression of HER-2 and HER-3 and the presence of ulceration in oromucosal tumors. This work confirms the expression of HER-2 and HER-3 in canine melanomas and suggests a putative association with negative prognostic parameters. Further studies are necessary to strengthen these data by increasing the samples size and combining pathological examinations with molecular biology in the investigation of EGFR family receptors.

## 1. Introduction

Canine malignant melanoma (CMM) is a relatively common and severe disease of dogs deriving from an uncontrolled proliferation of neural crest-derived melanocytes, which accounts for ~4% of all canine tumors and up to 7% of malignant tumors [1,2,3]. This neoplasm can arise in different anatomical areas, commonly the oral cavity (56%), lip (23%), skin (11%), and digit (8%), with other sites, including the eye, accounting for only 2% [4]. Although in all districts, these tumors derive from the same type of cells, sharing the embryonic derivation and the cellular function, the degree of malignancy is strictly related to the anatomical site in which they arise, probably because of distinct genetic alteration [3,5,6]. Particularly, oral and ungual melanomas display the most aggressive behavior, while cutaneous and ocular ones are often benign and rarely tend to metastasize [3]. Similar to veterinary medicine, melanoma is one of the most aggressive types of cancer even in the human counterpart. It is considered the most lethal cancer of the skin [7,8], but its highly heterogeneous nature is evident in humans as well, showing different behaviors in the function of the anatomical sites which, however, do not correspond to those observed in the canine counterpart at the same anatomical sites [5,9]. Furthermore, unlike in dogs, most melanomas are thought to be linked to ultra violet (UV) radiation, even if they are also frequently detected in non-UV-ray exposed sites [3]. The strong relevance of UV ray involvement in the pathogenesis of human malignant melanomas is also testified by the current World Health Organization (WHO) classification, classifying these tumors according to their UV exposure into the following three groups: melanoma arising in intermittently or chronically sun-exposed and melanoma arising in sun-protected skin [10]. The development and progression of melanoma are multigenic processes in which several pathways are involved. Although the underlying mechanisms are not yet fully understood, research is constantly moving forward, and a better knowledge of molecules and pathways can lead to more prognostic information and new therapeutic targets. Using this framework, investigations on several gene and protein expressions have been carried out, among which several studies have focused on human epidermal growth factor receptor (HER) family members [11]. The HER family comprises a series of transmembrane receptors, namely, EGFR (HER-1/HerbB-1), HER-2/HerbB-2, HER-3/HerbB-3, and HER-4/HerbB-4, located on the surface of cell membranes. These receptors are made up of three regions as follows: an extracellular epidermal growth factor (EGF) ligand domain, a transmembrane domain, and a cytoplasmic domain with tyrosine kinase activity [12]. EGF-binding-induced activation leads to molecular conformational changes, the homo- or heterodimerization of receptors, the stimulation of intracellular tyrosine residue, and the activation of several signaling pathways involved in cellular proliferation and differentiation [12,13,14]. Previous studies suggested that the HER family is also involved in tumor progression and that overexpression of HER family members can be associated with tumor progression and poor prognosis [15]. Nowadays, HER-2 is a well-established marker for human breast cancer (HBC) since its overexpression is observed in 20–30% of tumors and it is strongly correlated with poor prognosis [16,17]. On the other hand, the role of HER-3 in HBC progression is still under investigation. Some authors suggested that subjects with HER-3 overexpression showed a better prognosis [18,19], while others reported poor prognosis for these patients [20,21]. In the last decades, the expression of these receptors has been investigated in several types of tumors, but studies on melanomas are still fragmentary and controversial, both in human and veterinary medicine. This work aims to study HER-2 and HER-3 expression in canine melanomas and investigate their putative association with negative prognostic factors. 

## 2. Materials and Methods

### 2.1. Animals and Tissue Samples

Thirty-seven formalin-fixed paraffin-embedded (FFPE) melanomas were retrieved from the Tumor Registry of the Department of Veterinary Sciences at the University of Pisa. Among these, 18 were cutaneous melanomas excised from different areas of the skin, 16 were oral melanomas originating from the oral region, and 3 were digital melanomas arising in the digital area. All the samples came from solitary nodules that were surgically excised from dogs undergoing no other treatments except surgery. For each sample, breed, age, and sex were also recorded.

### 2.2. Histopathology

Four μm thick sections from each sample were cut and stained with Mayer’s hematoxylin and eosin (H-E) for histopathology examination under a light microscope. Histological classification of melanomas as epithelioid, fusiform, or mixed form was carried out according to the guidelines of the World Health Organization system [22]. For each case, the presence of ulceration was assessed, and the mitotic count was recorded as the number of mitoses in 2.37 mm^2^ [23]. Furthermore, a mean number ≥4 for oral lesions and ≥3 for cutaneous and digital ones was considered a high mitotic count [23]. Samples were then categorized into two groups as follows: samples with a low mitotic count and samples with a high mitotic count. 

### 2.3. Immunohistochemistry

Immunohistochemical analysis was carried out to confirm the diagnosis of melanoma and to assess the expression of HER-2 and HER-3 receptors. In the first case, a monoclonal mouse anti-Melan A (IS633, Dako, Glostrup, Denmark clone A103, dilution 1:100) and a monoclonal mouse anti-Melanoma Antigen (PNL2, 33-528-100, ProSci, Poway, CA, USA, dilution 1:100) were used. To evaluate the expression of the two members of the epidermal growth factor receptor (EGFR) family, a monoclonal rabbit anti-HER 2 (A048529-2, Dako, Glostrup, Denmark, dilution 1:50) and a polyclonal rabbit anti-HER 3 (BSB263, Bio SB, Santa Barbara, CA, USA, clone RBT-HER3, dilution 1:50) were employed. After deparaffination in xylene and rehydration in graded alcohol, before starting immunohistochemistry IHC procedures, pigmented lesions underwent a supplementary phase of bleaching by incubating them in 6% diluted hydrogen peroxide overnight at room temperature [24]. Briefly, immunohistochemical protocols included an antigen retrieval phase performed in a microwave with citrate buffer pH 6.0, boiling the sections for 4 min at 1000 W and 12 min at 200 W, followed by endogenous peroxidase activity blocking by incubating sections in Bloxall Blocking solution (SP-600, Vector, Burlingame, CA, USA) for 10 min. Ultra V Block (Thermo Fisher Scientific, Fremont, CA, USA) was employed to block nonspecific bindings for 5 min, and then sections were incubated overnight at 4 °C with the specific primary antibody. The following day, the sections were incubated with the secondary antibody (Biotinylated antibody universal, Anti-mouse/rabbit IgG, made in Horse, Vector Labs, Inc., Burlingame, CA, USA) for 20 min and then with streptavidin–biotin–peroxidase complex (Horseradish Peroxidase Streptavidin, Vector Labs, Burlingame, CA, USA) for a further 20 min. Finally, chromogen diaminobenzidine (Impact DAB, Vector Labs, Burlingame, CA, USA) was employed to develop the colorimetric reaction and Mayer’s hematoxylin to counterstain. The sections were then observed under a light microscope.

Positive and negative controls were supplied for each immunohistochemical analysis. Negative controls were performed by replacing the primary antibody with a species-matched unrelated antibody. For the positive controls, a canine mammary tumor already known to express the markers was employed for anti-HER-2 and HER-3 antibodies and, for anti-Melan A and anti-PNL-2, a canine amelanotic melanoma already known to express Anti-Melan-A and anti-PNL-2 antigens for these two markers. 

Anti-Melan-A and anti-PNL-2 antigens were considered positive when more than 10% of neoplastic cells showed cytoplasmic labeling. 

As for other histotypes of tumors [25], only the membranous pattern of the expression of HER-2 was taken into consideration, and it was categorized according to the following 4-point score adapted by [26]:

- Score 0: very weak incomplete membrane labeling in less than 10% of cells.
- Score 1+: very weak incomplete labeling in more than 10% of cells.
- Score 2+: weak or moderate incomplete membrane labeling in more than 10% of cells. or strong complete labeling in less than 10% of cells.
- Score 3+: strong complete membrane labeling in more than 10% of cells.

Different from the HER-2 evaluation, nuclear and non-nuclear (membranous/cytoplasmic) patterns of expression were separately considered in the HER-3 evaluation [25]. Nuclear expression was defined as nuclear immunolabeling of HER-3-positive samples and categorized into three staining patterns [27] as follows:

- Negative: (0, 0% labeled nuclei);
- Low: (1+, 1–17% labeled nuclei);
- Medium: (2+, 18–35% labeled nuclei);
- High: (3+, <35% labeled nuclei).

Non-nuclear HER-3 expression was defined as the expression of the HER-3 receptor in the cellular membrane or cytoplasm rather than in the nucleus. It was categorized by attributing a score from 0 to 4 to the labeling intensity of a minimum of 30% of tumoral cells as follows [26]:

- Score 0: no labeling in more than 70% of cells;
- Score 1+: weak label intensity in a minimum of 30% of cells;
- Score 2+: moderate label intensity in a minimum of 30% of cells;
- Score 3+: strong label intensity in a minimum of 30% of cells.

Scores 0 or 1+ were considered irrelevant, while scores 2+ or 3+ were considered positive. Consequently, samples were categorized into two groups according to their expression as follows: (1) samples with low score expression of receptors (score 0 or score 1), and (2) those with high score expression of receptors (score 2 or score 3). 

### 2.4. Statistical Analysis

Associations between variables were investigated through Chi-square or Fisher’s exact tests (*p*-value < 0.05) using the SPSS 11.0 program.

## 3. Results

The 37 melanomas included in this study came from 19 males and 17 females (in one case, the gender was unknown) with a mean age of 10.6 ± 3.4, ranging from 2 to 17 years old. Twenty-two subjects were mixed breeds, while, among the pure breeds, the Golden Retriever was the most representative (*n* = 3), followed by Bernese Mountain dog (*n* = 2), Dachshund (*n* = 2), Shar-pei (*n* = 2), and one subject each for each following breeds: American Pitbull Terrier, Labrador Retriever, Corsican, German Shepherd, Hungarian Pointer, ad Poodle. 

**Figure 1:**
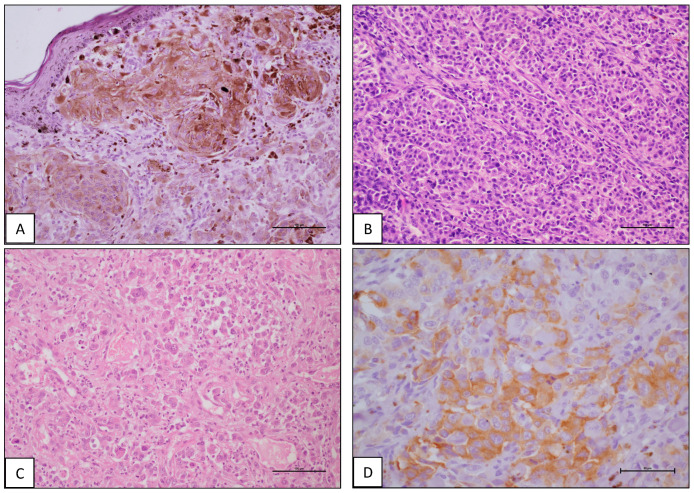
Canine malignant melanomas. (**A**) Cutaneous epithelioid malignant melanoma. H-E, scale bar 100 μm (**B**) Oromucosal epithelioid melanoma. H-E, scale bar 100 μm. (**C**) Cutaneous fusiform melanoma. H-E, scale bar 100 μm. (**D**) Oromucosal epithelioid melanoma. IHC with anti-Melan A. Arrows indicate positive cells. H-E counterstain, scale bar 50 μm.

From the 37 melanomas recruited, 18/37 (48.6%) arose in cutaneous areas, 16/37 (43.2%) in the oromucosal area, and 3/37 (8.10%) were taken from the footpad/digital area. According to the guidelines of the World Health Organization system (Goldschmidt and Hendrick, 2008), cutaneous samples were classified as epithelioid (10/18), fusiform (2/18), and mixed melanomas (6/18); oromucosal samples were classified as epithelioid (10/16), fusiform (3/16), and mixed melanomas (3/16); and digital samples as epithelioid tumors (2/3) and one mixed melanoma. Ulceration was observed in 20/37 samples, with four cases coming from the cutaneous area, 13 melanomas from the oromucosal area, and three samples from the digital area. A high mitotic count was observed in 4/18 cutaneous melanomas, 11/16 oromucosal melanomas, and one digital melanoma (Table 1). All the samples were positive for IHC using the anti-Melan A or the Anti-Melan Antigen PNL2 (Figure 1D). 

**Figure 2:**
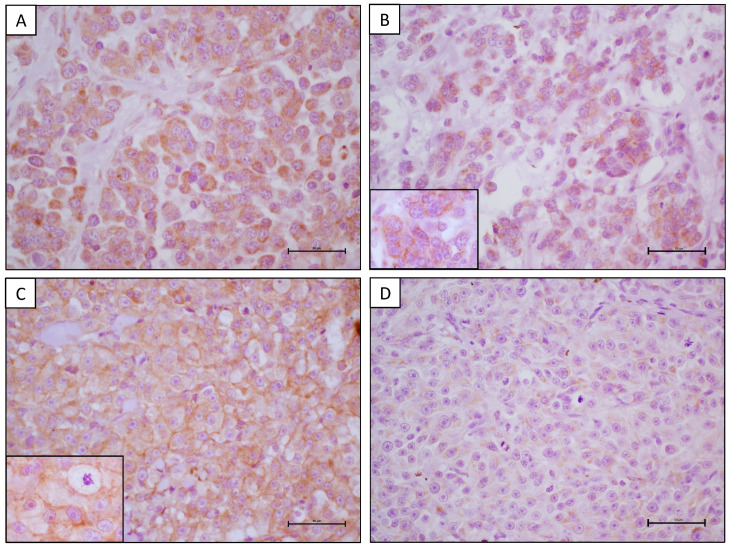
Expression of HER-2 (**A**–**C**) and HER-3 (**B**–**D**) in canine malignant melanomas. (**A**) Strong incomplete membranous HER-2 staining in more than 10% of cells and complete HER-2 staining in less than 10% of cells (score 2+). IHC with anti-HER-2 antibody, scale bar 50 μm. (**B**) Moderate cytoplasmic HER-3 staining in a minimum of 30% of cells (score 2+). Insert: Strong, incomplete membranous HER-3 staining in some cells. IHC with anti-HER-3 antibody, scale bar 50 μm. (**C**) Strong, complete membranous HER-2 staining in more than 10% of cells (score 3+). Insert: Particularly strong, incomplete membranous HER-2 staining in neoplastic cells showing mitosis and atypia. IHC with anti-HER-2 antibody, scale bar 50 μm. (**D**) Weak cytoplasmic HER-3 staining in a minimum of 30% of cells (score 1+). IHC with anti-HER-3 antibody, scale bar 50 μm.

HER-2 expression was observed in 18/37 (48.6%) samples (Figure 2A,C), including 9/18 (50%) cutaneous melanomas, 8/16 (50%) oromucosal melanomas, and one-third (33.3%) of the digital melanomas. Nuclear expression was absent in all samples. Non-nuclear HER 3 expression was evident in 7/37 (18.9%) samples (Figure 2B,D), including 3/18 (16.7%) cutaneous melanomas, 3/16 (18.8%) oromucosal melanomas, and one-third (33.3%) of the digital melanomas. A significant expression of HER 2 (score 3+) was recorded in 6/37 (16.2%) melanomas, including 3/18 (16.7%) cutaneous melanomas and 3/16 (18.8%) oromucosal melanomas (Figure 2C). Contextual expression of both receptors was observed in 5/37 (13.5%) cases as follows: 3/18 (16.7%) cutaneous melanomas, two (12.5%) oromucosal melanomas, and one-third (33.3%) of the digital melanomas (Figure 2A,B). There was no association between HER-2 or HER-3 expression and the various histotypes. The histopathological features of samples in combination with HER-2 and HER-3 status are reported in Table 2. In cutaneous melanomas, a high score expression of HER-2 and HER-3 was mainly associated with samples without ulceration and a low mitotic count, but this evidence was not significant. Conversely, in oral melanomas, a high score expression of HER-2 was associated with ulceration (even if not significative) and mitotic count (*p* < 0.05), and the same trend was observed for samples expressing HER-3 (*p* < 0.05 both for ulceration and mitotic count). In the digital melanomas, only one case expressed HER-2 and HER-3 with a high score, and this sample was ulcerated and had a high mitotic count. 

## 4. Discussion

HER-2 and HER-3 are members of the ErbB family of receptors tyrosine kinase involved in essential cellular functions, like proliferation, cell migration, metabolism, and survival [28]. The complex interactions among these receptors and, eventually, between them and their extracellular ligands result in homodimerization or heterodimerization with another member of the HER family and in the activation of different pathways, primarily the PI3K/AKT, MAP kinase, and JAK/STAT pathways [11,28]. When dysregulated, they can promote disordered proliferation, invasion, and unchecked cell survival, leading to the development of cancer. Aberration in signals may increase homo- or heterodimerization and constitutively active kinase activity [29]. Overexpression of these genes may be associated with tumor progression and poor outcome [11]. 

In human oncology, an association between ErbB gene overexpression and some malignancies has been reported, including breast cancer [30,31] but also prostate [32] and lung [33] neoplasms, gastric and colorectal cancer [34], and malignant melanoma [28,35]. For these reasons, their expression is often considered a poor prognostic factor. HER-2 overexpression has been associated with breast cancer as well as other solid tumors, such as prostate, ovarian, lung, gastric and colorectal cancer, and malignant melanoma. In some of these tumors, HER-2 overexpression has been associated with a worse prognosis [35]. The veterinary literature on ErbB family gene expression is extensive. The expression of epidermal growth factor receptors has been reported in several cancer types, including canine mammary carcinoma [25], gastric epithelial tumors [36], urothelial neoplasms [37,38], ovarian tumors [39], osteosarcoma [40], lung cancer [41], anal sac gland tumors [42] and feline mammary tumors [43,44], endometrial tumors [45], and pulmonary carcinoma [46]. Despite melanoma being among the first tumors in which EGFR is suggested to be a metastatic marker [47], little is known about ErbB receptors in this cancer, and the literature about this issue is sometimes controversial. In human medicine, EGFR expression in metastases from primary cutaneous melanomas has been suspected to have a prognostic significance. Alteration in the gene copy number of EGFR has been related to poor survival in cutaneous melanoma [47], and HER-2 overexpression has been described in thick cutaneous melanomas (thickness ≥ 10 mm), even if without proven prognostic significance [35]. On the contrary, HER-3 expression in primary melanoma, as well as in metastasis, has been associated with cell proliferation, tumor progression, and reduced patient survival, being evaluated as a negative prognostic marker. Similarly, little is known about the expression of epidermal growth factor receptor expression and its relevance as a prognostic marker in veterinary medicine. Veloso et al. [26] reported HER-2 membranous expression in 50% of oral tumors and 25% of skin tumors and HER-3 cytoplasmic expression in 18% of oral melanomas and 6% of cutaneous neoplasms. Our results overlap with those of Veloso et al. for oral melanomas since we described HER-2 and HER-3 expression in 50% and 18.8% of oral samples, respectively. In contrast, we observed a high percentage of HER-2 and HER-3 expression in cutaneous melanomas; particularly, HER-2 expression was observed in 50% of cutaneous samples and HER-3 expression in 16.7% of them. Moreover, in contrast to Veloso et al. [26], who did not report samples with a score of 3+, we found strong complete membrane labeling in more than 10% of cells (corresponding to a score of 3+) in five samples including three cutaneous and two oral melanomas. Furthermore, unlike Veloso et al., 2020, we also observed membranous expression of the HER-3 receptor in our melanoma samples, as already described in the literature in other canine tumors [25], but we did not find HER-3 nuclear expression. Despite this, at least in this preliminary evaluation, we decided to adopt only one score for HER-3 evaluation without distinguishing cytoplasmic and membranous labeling since, in our samples, the two patterns were very often contextually present. HER-2 expression was considered a negative prognostic factor in many tumor types; however, its expression in melanomas remains controversial both in human and veterinary medicine. On the one hand, many human studies have reported HER-2 overexpression very rarely in their samples [48,49,50], on the other hand, even when overexpression was reported, its role as a poor prognostic indicator still remained unclear. Our preliminary results show that in oral melanomas, HER-2 expression is significantly associated with a high mitotic count, and a similar association, even if not statically significant, was observed for ulceration, which is considered a negative prognostic factor [51,52]. This evidence, together with the correlation between the membranous expression of HER-2 and the presence of emboli in oral tumors seen by Veloso et al., 2020, points out the suspicion that the expression of this receptor might be implicated in the malignant progression of oral melanoma. The evaluation of HER-3 expression deserves a separate discussion. In our oral samples, HER-3 expression was statistically associated with ulceration and showed a putative association with a high mitotic count like HER-2, probably suggesting a possible role as a negative prognostic factor as well; however, the literature about HER-3 expression in canine tumors is still scarce. HER-3 expression seems to be relevant in predicting the biological behavior of neoplasms; however, the literature is controversial. It was reported that HER-3 membranous expression was a marker of poor prognosis in human melanoma [53], while HER-3 nuclear expression was negatively associated with proliferation index in canine melanoma [26]. On the contrary, in canine mammary tumors, HER-3 nuclear expression was associated with high histological grade and lymphatic invasion, while non-nuclear expression was higher in non-malignant tumors [25]. These incongruences, and the need to confirm the trends herein described, indicate the urgent need for more samples and more studies on these receptors. Nevertheless, we found a high HER-3 expression in tumors displaying negative prognostic indicators, suggesting the role of this marker in determining the malignancy of the neoplasm. Canine melanoma is a spontaneously occurring common disease, accounting for 4% of all canine tumors [2]. It can arise anywhere on the body surface, but it is common as oral, digital, and cutaneous tumors [52]. Depending on the location, the biological behavior of malignant melanoma is different. Oral melanoma is the most common oral tumor in dogs, and, together with neoplasms arising in the foot pad, it is considered very aggressive and frequently metastatic [2,52], while cutaneous melanomas represent the relatively benign counterpart of this neoplasm in dogs [52]. Even when treated with aggressive therapy like surgery, radiation therapy, and chemotherapy, metastasis is a common sequela of malignant melanoma both in humans and dogs since the response rate to chemotherapy is very low and the prognosis is often unfavorable [2]. Based on the evidence that ErbB receptor amplification and mutation might be implicated in several neoplastic diseases, in the last few years, new potential therapeutic strategies aiming to target these receptors have been proposed. The hypothesis that ErbB receptors might be involved in melanoma carcinogenesis paves the way for the use of monoclonal antibodies targeting the extracellular domain of these receptors and tyrosine kinase inhibitors. Given the multiple similarities summarized in [54], canine melanoma is nowadays considered the best translational model for comparative genetic and therapeutic applications in human melanoma. For this reason, further studies on prognostic markers in canine melanomas could have positive implications not only in veterinary but also in human medicine. 

This research is configured as a preliminary study on this understudied field. In the future, some progress could be made by collecting more samples, examining the expression of each receptor of ErbB family and interactions among the various receptor classes, studying further parameters and markers and correlating them with ErbB receptor expression, and trying to collect fresh samples in addition to FFPE ones to investigate not only immunohistochemical expression but also gene amplification.

## 5. Conclusions

This preliminary study confirms the expression of HER-2 and HER-3 receptors in canine melanoma and suggests a putative association among them and negative prognostic factors. Given the increasing importance of immunotherapy in the fight against cancer and the numerous similarities between canine and human melanoma, further studies are encouraged to investigate the role of these receptors in the neoplastic progression of this kind of tumor. 

## Figures and Tables

**Table 1:** Histopathological features of melanomas classified according to their prevalent pattern.

		Ulceration		Mitotic Count		Her 2		Her 3		Tot
		Yes	No	High	Low	0–1	2–3	0–1	2–3	
**Cutaneous melanoma**	**Epithelioid**	2	8	4	6	5	5	8	2	
	**Fusiform**	0	2	0	2	1	1	2	0	
	**Mixed**	2	4	1	5	4	2	6	0	
	**tot**	4	14	5	13	10	8	16	2	18
**Oromucosal melanoma**	**Epithelioid**	7	3	7	3	5	5	9	1	
	**Fusiform**	3	0	2	1	2	1	1	2	
	**Mixed**	3	0	2	1	1	2	3	0	
	**tot**	13	3	11	5	8	8	13	3	16
**Digital melanoma**	**Epithelioid**	2	0	1	1	1	1	1	1	
	**Mixed**	1	0	0	1	1	0	1	0	
	**tot**	3	0	1	2	2	1	2	1	3

**Table 2:** Features of samples classified according to HER-2 and HER-3 score of expression. * *p* < 0.05.

		Cutaneous Melanoma				Oromucosal Melanoma				Digital Melanoma			
		HER-2		HER-3		HER-2		HER-3		HER-2		HER-3	
		0–1	2–3	0–1	2–3	0–1	2–3	0–1	2–3	0–1	2–3	0–1	2–3
**Ulceration**	**yes**	3	1	4	0	5	8	10 *	3	2	1	2	1
	**no**	6	8	11	3	3	0	3	0	0	0	0	0
**Mitotic count**	**high**	1	4	5	0	3	8 *	8	3	1	1	1	1
	**low**	8	5	10	3	5	0	5	0	1	0	1	0
**tot**		9	9	15	3	8	8	13	3	2	1	2	1
		18		18		16		16		3		3	

## Data Availability

The data presented in this study are available in this article.
